# Cichlid Evolution: Lessons in Diversification 2012

**DOI:** 10.1155/2012/349485

**Published:** 2012-09-18

**Authors:** Stephan Koblmüller, R. Craig Albertson, Martin J. Genner, Kristina M. Sefc, Tetsumi Takahashi

**Affiliations:** ^1^Department of Zoology, Karl-Franzens-University Graz, Universitätsplatz 2, 8010 Graz, Austria; ^2^Department of Biology, University of Massachusetts, Amherst, Springfield, MA 01002, USA; ^3^School of Biological Sciences, University of Bristol, Woodland Road, Bristol BS8 1UG, UK; ^4^Graduate School of Science, Kyoto University, Kitashirakawa-Oiwake, Sakyo, Kyoto 606-8502, Japan

This is the second special issue on cichlid evolution hosted by the International Journal of Evolutionary Biology. Once more, we are overwhelmed by the vivid responses to our call for contributions, and thank the authors for their great work. The thirteen papers in this issue, including two reviews, span geographically from Africa to South America and address a wide variety of evolutionary topics including speciation and hybridization, phenotype evolution, and reproductive behaviour. Papers are summarized below in the order in which they appear in this special issue.

**Linking Diversification to the Physical Environment**

The East African cichlid radiations represent the most spectacular vertebrate radiations known to date, and a multitude of studies have sought to elucidate the phylogenetic relationships among extant East African cichlid lineages. The East African rift valley is also extremely dynamic from a geological perspective, and numerous studies have aimed at clarifying the geologic, hydrologic, and climatic history of this region. However, while these fields have enormous potential to inform one another, a uniting synthesis combining evidence from studies on geology/hydrology/climatology and cichlid phylogeny/phylogeography has been lacking. In their review “*The impact of the geologic history and paleoclimate on the diversification of East African cichlids,*” P. D. Danley et al. provide an up-to-date synthesis of the geologic history and paleoclimate of the East African Great Lakes (and surrounding water bodies) and the evolution of the region's endemic cichlid species assemblages. They discuss how tectonic processes and climatic changes changed the hydrological systems in East Africa and affected the diversification of cichlid fishes. Thus, their review links data on cichlid diversity with the highly variable geological and paleoclimatic history of East Africa and serves as an important reference for the impact of geological, paleoecological, and paleoclimatic factors on biogeography.

**Potential Roles for the Visual System in Promoting Adaptive Radiation**

Animals differ dramatically in their ability to the perceive signals in their natural environment, and during the last ten years we have gained a much better understanding of how closely related species differ in both their signalling and receiving abilities during information transfer. In cichlids, the role of spatial environmental variation in driving natural selection on these sensory traits has become increasingly appreciated, and it has been proposed that such “sensory drive” may even have promoted speciation through sexual selection in the Lake Victoria cichlid radiation. In their thought-provoking review “*An evaluation of the role of sensory drive in the evolution of Lake Malawi cichlid fishes,*” A. R. Smith et al. consider whether this model of evolution could also help to explain the evolution of remarkable cichlid species richness in Lake Malawi. They explore three aspects of the hypothesis. First, they ask if the broad light spectrum of the relatively clear waters of Lake Malawi is capable of driving strong natural selection on visual systems, as it has been shown to be the case in Lake Victoria. Second, they consider the extent of variation in visual perception present both within and among populations of Lake Malawi cichlids, with a specific consideration of the relative roles of functional genetic differences and the extent of developmental plasticity in determining adult visual phenotypes. Finally, they consider if links are present between male courtship colours and the ability of females to perceive those traits. On balance, the authors conclude that sensory drive is unlikely to be a major force in the evolution of Lake Malawi cichlids under present environmental conditions. However, it is also clear from the review that considerably more work is required to understand how cichlid sensory ecology is related to the process of adaptive radiation.

**Determining the Effects of the Visual Environment on Coloration**

In Lake Victoria visual environments are sufficiently diverse to provide divergent signalling conditions and create associations between sensory systems, signals, and environmental variation. In their study “*Species-specific relationships between water transparency and male coloration within and between two closely related Lake Victoria cichlid species,*” R. F. Castillo Cajas et al. demonstrate that the effects of visual environment on cichlid coloration can differ between closely related species, and, both within and across species, between body regions. While saturation and hue of the yellow/red body coloration of *Pundamilia nyererei* varied between populations in a way to enhance conspicuousness under local conditions, this was not apparent in the bluish body coloration of *P. pundamilia*. Different depth distributions or different sexual selection regimes in the two species may be responsible for species-specific responses to heterogenic signalling environments. In contrast, covariation of anal fin spot coloration with environmental conditions was congruent between species, and variation of the red coloration at the dorsal fin lappets of both species followed no consistent pattern. The study offers an illuminating glance on the complexity of the interactions among the multifarious influences and constraints acting on colour pattern evolution.

**Divergence and Genetic Basis of Internal Bone Geometry**

Beyond their rich colour variation, cichlids exhibit unparalleled levels of ecomorphological diversification, and this phenomenon has been extensively studied and characterized from the standpoint of external craniofacial bone morphology. In contrast, relatively little is known about whether biomechanically relevant shifts in internal bone architecture have accompanied this adaptive radiation. In “*More than meets the eye: functionally salient changes in internal bone architecture accompany divergence in cichlid feeding mode,*” R. C. Albertson and coauthors used **μ**CT analysis to show clear differences in the internal anatomy and load-bearing function of craniofacial bone in species that occupy distinct foraging niches. Moreover, a mapping experiment was used to characterize the genetic architecture of bone biomechanics. These results shed new light on the evolutionary diversification of feeding architecture in cichlids and highlight the importance of internal skeletal geometry in studies of adaptive radiations.

**The Feasibility of Hybridization in Divergent Species: A Genomic Perspective**

Hybridization is increasingly recognized as a positive force in the evolutionary diversification of cichlids. In “*Analysis of the meiotic segregation in inter-generic hybrids of tilapias,*” E. Bezault et al. analyzed meiotic segregation in the hybrid genomes of two divergent tilapia species, *Oreochromis niloticus* and *Sarotherodon melanotheron*. The authors found that in reciprocal F2 and backcross progeny patterns of segregation were similar to those of both parental species. These results provide important insights into genome evolution and the roles for hybridization in promoting cichlid evolution.

**Hybridization and Diversification in Lake Malawi Cichlids**

The East African lacustrine cichlid radiations have been particularly shaped by introgressive hybridization and even hybrid speciation. Even the origin of whole adaptive radiations via ancient hybridization has been repeatedly postulated. In their study “*Extensive introgression among ancestral mtDNA lineages: Phylogenetic relationships of the Utaka within the Lake Malawi cichlid flock*,” D. Anseeuw et al. provide evidence for past hybridisation among divergent Lake Malawi cichlid lineages and thus further highlight the importance of interspecific gene flow for shaping the evolutionary history of East African cichlid fishes. Specifically, highly divergent mtDNA lineages have been found in several species of the Utaka, an informal group of Lake Malawi cichlid species. Nuclear data, on the other hand, did not show comparable patterns of intraspecific divergence. The authors conclude that the observed discrepancy between mtDNA and nuclear DNA is best explained by introgression of divergent mtDNA into ancestral representatives of the Utaka.

**Deepwater Communities Are Not Shaped by Introgression**

Several molecular phylogenetic studies inferred hybridization among littoral cichlid species in Lake Tanganyika, a phenomenon typically attributed to recurrent climate-driven lake level fluctuations altering shoreline and habitat structure. In “*Evolutionary history of Lake Tanganyika's predatory deepwater cichlids,*” P. C. Kirchberger et al. show that in contrast to the predominantly littoral Lake Tanganyika cichlid lineages studied so far, the evolutionary history of the large predominantly piscivorous species of the tribe Bathybatini appear to have been not affected by introgressive hybridization and that inconsistencies between nuclear and mitochondrial phylogenetic trees are likely due to ancient incomplete lineage sorting. Their findings are consistent with the hypothesis that lineages evolving in the weakly structured deepwater habitat would develop stronger reproductive isolation than the often allopatric lineages in the highly fragmented littoral. However, whether this lack of hybridization among deepwater taxa is representative of a general pattern typical for cichlid lineages inhabiting a weakly structured habitat or applies to just particular lineages remains to be tested by analysing additional openwater and deepwater lineages.

**Genetic Evidence in Support of Divergence between Sympatric Colour Morphs**

A key issue in cichlid taxonomy is what characters we should use to delimit species. In African haplochromine cichlids, for example, male breeding colours appear to be effective indicators of species boundaries, at least among sympatric populations. In the monotypic Lake Tanganyika genus *Cyathopharynx* (tribe Ectodini) two distinct colour morphs occur sympatrically around most of the lake; however they have been treated as a single species in the scientific literature. In “*Genetic and morphological evidence implies existence of two sympatric species in Cyathopharynx furcifer (Teleostei: Cichlidae) from Lake Tanganyika*," T. Takahashi and M. Hori convincingly demonstrate that these two distinct morphs actually represent two distinct species.

**Genetic Evidence *Fails* to Support Divergence between Sympatric Colour Morphs**

In a similar study, “*Deep phylogenetic divergence and lack of taxonomic concordance in species of Astronotus (Cichlidae),*” O. P. Colatreli and coauthors tested for genetic differences between sympatric and allopatric populations of *A. ocellatus* and *A. crassipinnis,* two Amazonian cichlids from the “oscar” genus that can be distinguished based on adult colour patterning. Their results showed no clear evidence of genetic differences among the sympatric colour forms. The analyses did however uncover the presence of five very substantially divergent allopatric lineages that may be new species. Further research would help to confirm this interpretation of the data and will require more genetic, morphological, and behavioural data. Oscars are among the most iconic of all cichlids, yet O. P. Colatreli and coauthors have shown a good deal of work is still required to catalogue the true diversity of their genus. This equally applies to many other genera of Neotropical and African cichlids.

**Phylogeography of a Rapidly Dispersing Species**

In “*Phylogeographic diversity of the lower Central American cichlid Andinoacara coeruleopunctatus (Cichlidae),*” S. S. McCafferty et al. used phylogeography to disentangle the relative contributions of historical and ecological processes in determining the biogeography of the lower Central American cichlid species *Andinoacara coeruleopunctatus*. Specifically, the authors used mtDNA sequence and RFLP data to test the hypothesis that, given its high dispersal capabilities, phylogeographic patterns in *A. coeruleopunctatus* will differ from those of other species. Significant phylogeographic structure was observed across lower Central American populations of *A. coeruleopunctatus*, and phylogeographic patterns were consistent with the rapid colonization and dispersal of this species following the rise of the Isthmus of Panama. This study provides insight into the processes that determine biogeography and underscores the value of studying species with distinct life histories and ecologies within this context.

**Exploring the Life History and Reproductive Behaviour of a Shell-Brooding Cichlid**

New details on the life cycle of a cichlid from Lake Tanganyika are provided by K. Ota et al. in their study, “*Alternative reproductive tactics in the shell-brooding Lake Tanganyika cichlid Neolamprologus brevis.*” Among their notable findings, the authors show that the distribution of this species across divergent habitats depends on their reproductive stage, and that they migrate to communal nests, that is, the shell patches assembled by another shell-brooding species, only temporarily for reproduction. Moreover, behaviours, body, and gonad sizes of males among shell patches provide evidence for a multimale polygynous mating system, and for the employment of sneaking as an alternative reproductive tactic in this lamprologine cichlid.

**Coordination of Spawning in the Absence of Visual Cues**

Another study in this issue focuses on the builder of these communal shell nests. The shell-brooding *Lamprologus callipterus* from Lake Tanganyika exhibits the most extreme sexual size dimorphism among cichlid fishes (males > females). Females spawn their eggs while inside gastropod shells, and males fertilize eggs from outside the shell. Although males of many cichlid species use visual stimulation to induce egg laying in their partners, pairs of this species cannot see each other during spawning. This obviously raises the question of how gamete release is synchronized between the sexes. In “*Spawning coordination of mates in a shell brooding cichlid,*" D. Schütz et al. inferred, based on field observations and laboratory experiments, that the male initiates the spawning sequence and that sperm release and egg laying are very well synchronised despite the limited communication possibilities during spawning. Females attempt to extend the egg laying period to increase the chance for parasitic males to participate in spawning in order to induce sperm competition. The authors also discuss the possibility that this exceptional spawning pattern reflects a conflict between the sexes.

**Cross-Species Amplification of Microsatellites**

The concluding paper of this issue is a technical note by E. Bezault et al., “*Microsatellites cross-species amplification across some African cichlids*,” which tested the applicability of a large panel of microsatellite markers in a total of 15, mostly tilapiine, African cichlid species, and provided the community with a powerful marker arsenal for further molecular genetic studies.

